# Guizhi Fuling wan for chronic pelvic inflammatory disease protocol: A protocol for systematic review and meta analysis

**DOI:** 10.1097/MD.0000000000023549

**Published:** 2020-12-18

**Authors:** Chunrong Wang, Jingyun Chen, Yanling Xiao, Qilin Shen

**Affiliations:** College of Basic Medicine, Chengdu University of Traditional Chinese Medicine, Chengdu, Sichuan, China.

**Keywords:** chronic pelvic inflammatory disease, Guizhi Fuling wan, meta-analysis, modified Guizhi Fuling wan, protocol, systematic review

## Abstract

**Background:**

Chronic pelvic inflammatory disease (CPID) is one of common diseases of department of gynaecology, point to female inside genital and circumferential organization to suffer from infection of all sorts of pathogenic bacteria and cause chronic inflammation sex disease, also cause one of main factors of infertile of female of childbearing age period. Due to its insidious onset, it is not easy to find out in the early stage. Therefore, it is difficult to obtain satisfactory curative effect by taking routine treatment with antibiotics. In recent years, TCM has made great strides in the treatment of chronic pelvic inflammation, a number of clinical studies have shown that Guizhi Fuling wan combined with antibiotics can significantly improve the clinical symptoms and enhance the therapeutic effect. Therefore, we intend to conduct a system review and meta-analysis to further clarify the effectiveness and safety of GZFLW for CPID.

**Methods:**

We will search each database from the built-in until September2020.The English literature mainly searches Cochrane Library, PubMed, EMBASE, and Web of Science, while the Chinese literature comes from CNKI, CBM, VIP, and Wangfang database. Simultaneously we will retrieval clinical registration tests and grey literatures. This study only screens the clinical randomized controlled trials (RCTs) about GZFLW for CPID to assess its efficacy and safety. The 2 researchers worked independently on literature selection, data extraction, and quality assessment. The dichotomous data is represented by relative risk (RR), and the continuous is expressed by mean difference (MD) or standard mean difference (SMD), eventually the data is synthesized using a fixed effect model (FEM) or a random effect model (REM) depending on whether or not heterogeneity exists. The clinical efficacy, pelvic effusion and mass were evaluated as the main outcomes. The serum interleukin-6 (IL-6), C-reactive protein (CRP), tumor necrosis factor (TNF)-α, erythrocyte sedimentation rate (ESR), erythrocyte specific volume was secondary outcomes. Finally, meta-analysis was conducted by RevMan software version 5.3.

**Results:**

This study will provide high-quality evidence for treatment of CPID with GZFLW in terms of effectiveness and safety.

**Conclusion:**

This systematic review aims to provide new options for GZFLW treatment of CPID in terms of its efficacy and safety.

**Ethics and dissemination:**

This study does not require ethical approval. We will disseminate our findings by publishing results in a peer-reviewed journal.

**OSF registration number:**

DOI 10.17605 / OSF.IO / R9NVT.

## 1 Introduction

Chronic pelvic inflammatory disease is a common reproductive system disease in gynecology, including endometritis, salpingitis, salpingoovarian abscess, pelvic peritonitis and so on.^[1]^ Pathogenic bacteria infection causes local tissue congestion, edema, inflammatory exudation, connective tissue hyperplasia, can cause menstruation, abnormal leucorrhea, chronic pelvic pain, infertility and other problems, with long course of disease, low cure rate, high recurrence rate, anti-infection, anti-inflammatory is the main treatment method.^[2]^ Tinidazole combined with levoxacin in the treatment of chronic pelvic inflammation can rapidly improve the clinical symptoms, shorten the improvement time of clinical symptoms, improve the hypercoagulable state of the body blood, reduce the inflammatory response, improve the therapeutic effect, and promote the patients disease prognosis.^[3]^ Although antibiotic treatment has certain effects, patients are prone to develop adverse reactions such as dizziness, nausea, abdominal pain and allergy after treatment, and are prone to develop drug resistance. Therefore, safety needs to be improved. At the same time, after the application of anti-inflammatory drugs, the disease is not easy to recover, relatively stubborn, and accompanied by a very high recurrence rate.

Traditional Chinese medicine for chronic pelvic inflammatory disease research in China has been long, Traditional Chinese medicine (TCM) believes that the disease is caused by the invasion of toxins caused by the deficiency of Qi and blood during the period or postpartum, belong to “ abdominal mass ” disease, most of them take “deficiency of the original standard and solid, deficiency and solid mixed” as the evidence, deficiency of the Yang as the basis, and dampness-stasis block“ as the standard, should been used and promoting blood circulation to remove blood stasis as the main principle. The treatment is to activate blood circulation and remove blood stasis, slow and eliminate ruffian block as the method, clinical with Guizhi Fuling wan with the disease added or reduced treatment. The compound of Guizhi Fuling wan was first recorded in Zhang Zhongjings Synopsis of Jingui Yaolue, which consists of cassia twig (Guizhi), poria cocos (Fuling), peony bark (Mudan) and (Shaoyao). Modern pharmacological studies have shown that the prescription contains paeoniflorin, paeoniflorin, cinnamic acid, and other components,^[4]^ and has various effects such as analgesia, anti-inflammatory, and immune regulation.^[5–9]^ Studies have shown that patients with cassia twig tuckahoe pills can strengthen ability of permeability, effectively inhibit blood capillary permeability, still can enhance the activity of protease dissolving, inhibition of connective tissue hyperplasia at the same time, effectively improve the patients microcirculation, increase blood flow, so as to improve the condition of pelvic blood anoxic, thus reduce the formation of collagen fibers, promoting tissue regeneration repair.^[10]^ In another clinical study, the experimental group with Guizhi Fuling wan showed significantly better TCM symptom score, erythrocyte deposition rate, serum HS-CRP, hemorheology indexes, gynecological ultrasound examination results, and total therapeutic efficiency.^[11]^

Therefore, no matter from a large number of animal experiments or clinical experiments, it has been proved that Guizhi Fuling wan has a very clear curative effect in the treatment of chronic pelvic inflammation. However, there is no systematic study on the efficacy and safety of Guizhi Fuling wan the treatment of chronic pelvic inflammation. In this study, meta-analysis was used to systematically evaluate the efficacy and safety of Guizhi Fuling wan in the treatment of chronic pelvic inflammation, so as to provide strong evidence-based medicine support for its clinical application.

## 2 Methods

### 2.1 Protocol and registration

The protocol has been registered on the Open Science Framework (OSF) platform (https://osf.io/r9nvt/), registration number: DOI 10.17605 / OSF.IO / R9NVT. This protocol was drafted and reported in accordance with the Preferred Reporting Items for Systematic Reviews and Meta-Analyses Protocols (PRISMA-P) guidelines.^[12]^ If there are any adjustments throughout the study, we will fix and update the details in the final report.

### 2.2 Ethics

We will not need individual data of each patient in the research as this is a systematic review. Therefore institutional review board approval and ethics committee is not needed. Our purpose is to publish the results in a peer-reviewed journal. The final results of the review will provide information about the safety and efficacy of GZFLW and its modified forms in the treatment of CPID to help clinicians make decisions on clinical practice.

### 2.3 Inclusion criteria

#### 2.3.1 Type of study design

The study only select clinical randomized controlled trials of GZFLW for CPID published in both Chinese and English. However, animal experiments, reviews, case reports, and non-randomized controlled trials are excluded.

#### 2.3.2 Participants

The patients of CPID must meet the diagnostic criteria established by the relevant diagnostic criteria in The Code for The Diagnosis and Treatment of Pelvic Inflammatory Disease (Revised edition)^[13]^ and the Guiding Principles for clinical Research on New Chinese Medicines,^[14]^ and the diagnosis was confirmed by B-ultrasound imaging tests. Patients with liver and kidney insufficiency, pregnant and lactating women, pelvic tuberculosis, gynecological malignant tumor, endometriosis and other gynecological diseases, autoimmune system and blood diseases are not included. No gender, race, nationality and comorbidity are limited.

#### 2.3.3 Interventions

The control group selected broad-spectrum antibiotics according to experience, and the experimental group combined with Guizhi Fuling wan or added or reduced, modified Guizhi Fuling wan on the basis of the control group. The dosage, dosage and treatment time of the 2 groups were not considered in this study. Studies involving acupuncture, moxibustion, massage, and other TCM prescription will be eliminated. Additionally, the authors are about to eliminate studies involving unfixed TCM prescriptions.

#### 2.3.4 Outcomes

The primary outcomes was evaluated according to the literature standard,^[15]^ Recovery: after 2 weeks of treatment, the patients clinical symptoms such as fatigue and lower abdominal pain all disappeared. Ultrasound examination showed no effusion or mass in the pelvic cavity, and no abnormality in the uterus and accessories. Obvious effect: after 2 weeks of treatment, symptoms such as fatigue and lower abdominal pain were significantly improved, and the reduction of pelvic effusion and mass reduction in ultrasound examination were ≥50%;Effective: After 2 weeks of treatment, symptoms such as fatigue, lower abdominal pain and other relief, ultrasound examination of the reduction of pelvic effusion and mass shrink <50%; no effect: 2 weeks after treatment, the patients clinical symptoms did not alleviate or even worse than before treatment. Total effective rate = (cure + obvious effect + effective rate)/total cases×100%.

Secondary outcomes included serum interleukin (IL-6), C-reactive protein (CRP), tumor necrosis factor (TNF)-α, erythrocyte sedimentation rate (ESR), and erythrocyte specific volume.

### 2.4 Search methods

#### 2.4.1 Electronic searches

Following databases will be searched: PubMed, MEDLINE, EMBASE, Cochrane Library, China National Knowledge Infrastructure (CNKI), Wanfang data, Chinese Scientific Journals Database (VIP), and China biomedical literature database (CBM). We will select the eligible studies published up to September 1, 2020. We adopt the combination of heading terms and free words as search strategy which decided by all the reviewers. Search terms: Guizhi Fuling wan, Guizhi Fuling capsule, modified Guizhi Fuling wan, Guizhi Fuling Pill, pelvic inflammation, chronic pelvicinflammatory disease. Taking PubMed as an example, the initial search strategy is shown in Table 1, which will be adjusted according to the specific database.

**Table 1:** Search strategy of the PubMed.

Number	Search terms
#1	Chronic pelvic inflammatory disease [Mesh]
#2	Chronic pelvic inflammatory disease [Title/Abstract] OR Chronic pelvic inflammatory diseases [Title/Abstract] OR The chronic pelvic inflammation [Title/Abstract] OR The chronic pelvic inflammations [Title/Abstract] OR Chronic pelvic inflammatory [Title/Abstract]
#3	#1 OR #2
#4	Erchen [Title/Abstract]
#5	Decoction [Title/Abstract]
#6	#4 AND #5
#7	randomized controlled trial [Publication Type]
#8	controlled clinical trial [Publication Type]
#9	Randomized [Title/Abstract]
#10	Randomly [Title/Abstract]
#11	#10 OR #11 OR #12 OR #13
#12	#3 AND #6 AND #11

#### 2.4.2 Searching other resources

At the same time, we will retrieve other resources to complete the deficiencies of the electronic databases, mainly searching for the clinical trial registries and grey literature about GZFLW for CPID on the corresponding website.

### 2.5 Data collection and analysis

#### 2.5.1 Selection of studies

**Figure 1:**
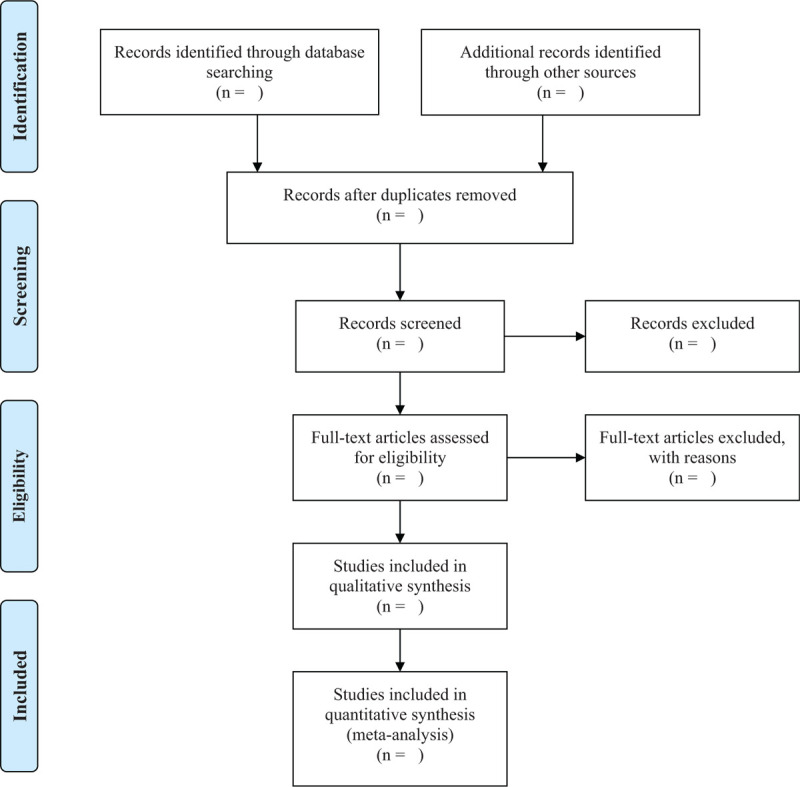
PRISMA flow diagram of the study selection process.

Import all literatures that meet the requirements into Endnote X8 software (Thomson Research Soft, Stanford, Connecticut). First of all, 2 independent reviewers (Chunrong Wang and Jingyun Chen) initially screened the literatures that did not meet the pre- established standards of the study by reading the title and abstract. Secondly, download the remaining literatures and read the full text carefully to further decide whether to include or not. Finally, the results were cross-checked repeatedly by reviewers. If there is a disagreement in the above process, we can reach an agreement by discussing between both reviewers or seek a third partys opinion (Yanling Xiao). Flow chart of the study selection (Fig. 1) will be used to show the screening process of the study.

#### 2.5.2 Data extraction and management

According to the characteristics of the study, we prepare an excel form for data collection before data extraction. Outcome indicators for eligible studies were independently extracted and filled in the data extraction form by 2 reviewers (Chunrong Wang and Jingyun Chen). If there is any argument, it can get an agreement by discussing through 2 reviewers or seek a third partys suggestion (Yanling Xiao). The main data extracted are as follows: title, author, year, fund source, sample size, age, sex, duration of disease, interventions, outcome measures, adverse reactions, etc. If you find something unclear in the study, you can contact the author of the communication directly for more detailed information. The above information was finally cross-checked by 2 reviewers.

#### 2.5.3 Assessment of risk of bias in included studies

The quality assessment of RCTs adopts the risk of bias (ROB) assessment tool provided by the Cochrane Handbook. The following 7 items, such as random sequence generation, allocation concealment, blinding of participants and personnel, blinding of outcome assessment, incomplete outcome data, selective outcome reporting, and other bias, are evaluated by 3 grades of “low bias,” “high bias,” and “unclear bias.” The discrepancies will get a consistent conclusion by discussing between both reviewers or seeking the third-party consultation.

#### 2.5.4 Measures of treatment effect

Different evaluation methods are selected according to the different efficacy indicators. For the dichotomous data, we will choose the effect scale indicator relative risk (RR) with 95% confidence interval (CI) to represent. While the continuous data is expressed as mean difference (MD) or standardized mean difference (SMD) with 95% CI depending on whether the measurement scale is consistent or not.

#### 2.5.5 Dealing with missing data

The reviewers will contact the first author or correspondent author via email or telephone to obtain missing data if the relevant data is incomplete. If the missing data is still not obtained in the above way, we can synthesize the available data in the initial analysis. Furthermore, sensitivity analysis will be used to assess the potential impact of missing data on the overall results of the study.

#### 2.5.6 Assessment of heterogeneity

Heterogeneity will be assessed by Chi-Squared test and *I*^2^test. If *I*^2^ < 50%, *P* > .1, we consider that no statistical heterogeneity between each studies and choose fixed effect model (FEM) to synthesize the data. If *I*^2^ ≥ 50%, *P* < .1, indicating that there is a statistical heterogeneity, the data are integrated by the random effect model (REM). In addition, due to differences in heterogeneity, we will conduct subgroup or sensitivity analysis to look for the potential causes.

#### 2.5.7 Data analysis

Review Manager software version 5.3 (The Nordic Cochrane Center, The Cochrane Collaboration, 2014, Copenhagen, Denmark) provided by the Cochrane Collaboration will be performed for data synthesis and analysis. The dichotomous data is represented by RR, continuous data is expressed by MD or SMD. If there is no heterogeneity (*I*^2^ < 50%, *P* > .1),the data are synthesized using a fixed effect model. Otherwise (*I*^2^ ≥ 50%, *P* < .1), a random effect model is used to analyze. Then subgroup analysis will be conducted basing on the different causes of heterogeneity. If a meta-analysis cannot be performed, it will be replaced by a general descriptive analysis.

#### 2.5.8 Subgroup analysis

If the results of the study are heterogeneous, we will conduct a subgroup analysis for different reasons. Heterogeneity is manifested in the following several aspects, such as race, age, sex, different intervention forms, pharmaceutical dosage form, dosage, treatment course.

#### 2.5.9 Sensitivity analysis

Sensitivity analysis is mainly used to evaluate the robustness of the primary outcome measures. The method is that removing the low-level quality study one by one and then merge the data to assess the impact of sample size, study quality, statistical method, and missing data on results of meta-analysis.

#### 2.5.10 Reporting bias

If there are >10 studies in the meta-analysis, the symmetry of the funnel plot will be assessed to examine publication bias, with results being interpreted cautiously.

#### 2.5.11 Grading the quality of evidence

In this systematic review, the quality of evidence for the entire study is assessed using the “Grades of Recommendations Assessment, Development and Evaluation (GRADE)” standard established by the World Health Organization and international organizations.^[16]^ To achieve transparency and simplification, the GRADE system divides the quality of evidence into 4 levels: high, medium, low, and very low. The GRADE profiler 3.2 will be employed for analysis.

## 3 Discussions

Chronic pelvic inflammatory disease is a chronic inflammatory disease that occurs in the internal genitalia and surrounding tissues such as ovaries, fallopian tubes, uterus, etc., and mostly occurs in women aged 20 to 35 years of childbearing age, with an incidence of about 40%.^[17]^ For the treatment of chronic pelvic inflammatory disease, currently western medicine widely USES antibiotic treatment, but long-term use of antibiotics will make the bacteria develop resistance, which is easy to cause treatment is not complete, disease relapse and other conditions, less than expected efficacy. Traditional Chinese medicine believes that the treatment of this disease to ”regulate Qi and activate blood circulation, remove blood stasis and remove dampness, tongluo pain", so choose Guizhi Fuling wan to treat chronic pelvic inflammatory disease. Modern studies have found that Guizhi Fuling wan not only has bactericidal, anti-inflammatory, anti-tumor, antipruritic, analgesic and other effects, but also can effectively improve blood circulation, enhance immunity and regulate the endocrine system.^[18]^

A number of clinical studies at home and abroad have shown that the application of antibiotics combined with Guizhi Fuling wan in the treatment of chronic pelvic inflammation can reduce the score of traditional Chinese medicine symptoms and improve the therapeutic effect. However, there has been no systematic review or meta-analysis to evaluate the therapeutic effect. Therefore, we conduct this systematic review to further evaluate the effectiveness and safety of GZFLW for CPID. Our aim is to provide more clinical evidence helping clinicians make decisions on clinical practice in CPID treatment.

## Author contributions

Chunrong Wang and Jingyun Chen made the same contribution to the research and design, and wrote the original draft of the protocol. Chunrong Wang has developed a search strategy. Chunrong Wang, Jingyun Chen and Yanling Xiao will conduct literature retrieval and collation. Chunrong Wang, Jingyun Chen and Qilin Shen will evaluate the risk of bias in the literature. Data analysis and article writing will be done by Chunrong Wang and Jingyun Chen. Qilin Shen, as the corresponding author, will be responsible for overseeing every process of the audit review to control the quality of the study. All the authors have approved the publication of the protocol.

**Data curation:** Qilin Shen.

**Funding acquisition:** Chunrong Wang.

**Investigation:** Qilin Shen.

**Project administration:** Yanling Xiao, Qilin Shen.

**Resources:** Qilin Shen.

**Writing – original draft:** Chunrong Wang.

**Writing – review & editing:** Jingyun Chen.
